# Some Likely Effects of Excessive Sickness Claims

**Published:** 1913-12-13

**Authors:** 


### NATIONAL INSURANCE ACT DEVELOPMENTS.

### Some Likely Effects of Excessive Sickness Claims.

In our issue last week we considered somewhat fully the important question of the valuation of the assets and liabilities of approved societies. The matter was treated with as little use as possible of technical terms in order that the fundamental principles underlying the methods employed by actuaries might be firmly grasped, for it is only when these principles are intelligently understood that it will be possible to follow the reports of the actuaries on the affairs of the approved societies. When, however, the principles are understood, it will be a comparatively easy matter to gauge from such reports the progress that is being made, and to what extent suggested changes are likely to affect the position of the societies. In concluding our article last week we drew attention to some grave words uttered by Sir Gerald Ryan—a high authority on such matters—on the subject of excessive sickness claims, and we now proceed to consider this aspect of the matter somewhat more fully. We shall also point out certain important effects that will be produced if such abnormally high sickness rates, as have been indicated, are found to be substantiated by fact throughout the country.

In this connection we look forward with a good deal of interest, not to say apprehension, to the report of the Committee appointed by the Government earlier in the year to inquire fully into this very question. It is understood that the Committee have completed their labours in so far as the collection of evidence is concerned. The matter is so vital to the interests of the approved societies, and through them to the insured persons generally, that it is to be hoped that their report will not long be delayed.

### Effects of a Permanent Alteration in the Rates of Sickness.

In order to concentrate our ideas, let it be assumed that in all respects, other than that of the rate of sickness, the estimates of the actuaries at the inception of the Act are exactly fulfilled. Furthermore, let us first consider the approved societies as a whole, and leave till later the more complicated question of the result of rates of sickness being permanently different in two or more apparently similarly constituted societies. A proper appreciation of the question, moreover, will only be gained if the fact, to which we drew special attention in our last issue, be fully grasped—namely, that the actuarial estimates in regard to claims for sickness benefit on the approved societies were based on the assumption, broadly speaking, that the sickness experience of the friendly societies before the days of National Insurance would be reproduced in the future.

Now, assuming that the original estimates with regard to sickness claims are exceeded, the question naturally arises, "What will be the effect on the societies?" It goes without saying that the immediate result will be a strain on the available funds, and probably a deficiency at the first valuation. But the matter has a deeper significance than this. It must be definitely decided, by those who have the power to judge, whether such abnormal sickness claims are merely a temporary fluctuation from the estimate, or whether they are likely to be a permanent feature of the experience of the societies.

This question in itself raises many important subsidiary points, chief among which is the necessity for ascertaining, if possible, to what main causes the excess sickness is due, as a careful study of these causes will probably reveal a considerable measure of information regarding the permanence, or otherwise, of the high sickness rate, and the possibility, or otherwise, of employing remedial measures to check this high rate. If the conclusion be reached that the excessive sickness claims is merely a temporary fluctuation, it would not be difficult, with a little immediate sacrifice on the part of the members of the societies, or by other means, to make good any deficiency produced.

Such temporary fluctuations from the expected sickness claims are to be anticipated, and in the ordinary course of things it would probably be found that an excess amount of claims and its consequent strain in one valuation period would be, to some extent, counterbalanced by a gain in a subsequent period.

### Insufficiency of Contributions.

If, however, the excess sickness is shown to be of a permanent character the matter is considerably more serious. Two important considerations here present themselves. It must be remembered that the original estimates as to sickness claims were used not only as the basis of the calculations to ascertain the necessary contributions, but also in the calculation of the initial reserves, by means of which persons above sixteen years of age are able to share in the benefits as though they were aged sixteen at entry.

If the original estimate is likely to be permanently exceeded to a degree greater than is covered by the margin for contingencies in the contributions it necessarily follows, if no saving can be effected in other directions, that such contributions will be insufficient. The position of the societies would then be difficult indeed. It would be absolutely necessary if the true financial position is to be shown for this permanently increased sickness rate to be given effect to in valuing the liabilities. This would have the obvious result of increasing the value of the liabilities. Since, however, the value of the contributions will remain unaltered, no change will take place in the value of the assets, and the net liability of the societies would be considerably increased. In respect, therefore, of a permanent increase in the sickness rate there would be at the first valuation, if true valuation methods are followed, virtually a double deficiency, one portion arising from excess sickness claims in the past, and the other arising from expected sickness claims in the future.

It is not our intention here to deal with the remedies to be applied to meet the situation indicated, but it may be remarked in passing that such remedies must in most cases take the form of an increased contribution or a reduction in benefit, either of which forms would necessarily be obnoxious to the ordinary member. There is thus substantial foundation for the fears expressed in our leading article of last week's issue, that the members may agitate for some relief to be obtained by a reduction of the remuneration to the doctors.

With regard to future members of the societies, these could not in fairness be admitted except at sufficient rates of contribution, as otherwise each member as he was admitted would cause a loss to the society.

### The Effect on the Reserves.

The second matter to which we would draw attention in connection with this hypothesis of a permanently increased rate of sickness is that of the effect produced on the "reserves" set up at the inception of the operation of the Act. These reserves were calculated on the basis of the sickness rates as originally estimated, and if any permanent increase in these sickness rates be experienced the amount of these reserves would have to be entirely re-calculated if they are to serve the purpose intended. The point is, of course, included by implication in our previous remarks, as these reserves are part of the assets that the societies hold against their liabilities which, as previously shown, would be increased. Any increase of the values would necessarily defer for a still longer period the time when the young persons will obtain the full advantage of the increased benefits promised under the Act, when the reserves have been liquidated.

Sufficient has now been said to show that, if there is any likelihood of a permanently high sickness rate, an extremely difficult situation will result, and a considerable anxiety will be given to those who act as advisers of the approved societies.

### The Formation of Class Societies.

We have, up to this point, purposely considered the societies as a whole. For valuation purposes however, the societies will not be treated in this manner, but will be dealt with either individually or in small groups, and it will be well to consider the question of excess sickness from the point of view of actual fact. One outstanding development of the powers conferred by the Act for the formation of approved societies has been that many societies have been formed whose members consist solely of persons engaged in a particular occupation. Thus societies have been formed in which membership is restricted to nurses, domestic servants, bank clerks, agricultural workers, etc., the main reason for the formation of these societies being the anticipation that persons engaged in these occupations will experience a lighter rate of sickness than the average insured person. Now, the rates of sickness which formed the basis of the National Insurance Act were derived from a general body of persons in which all occupations were combined. The self-selection of such persons, as above indicated by the formation of separate societies, will result somewhat unexpectedly in destroying the basic calculations. The withdrawal of these special classes from the main body would of itself, without other influences, cause an increase in the average rate of sickness of that main body, and we are at once face to face with the reasonable argument that in the majority of the societies the sickness rates will be permanently above the average, while in others they will be permanently below the average. Much depends, of course, on the actual extent to which the self-selection referred to has taken place, and on the extent to which the prognostications regarding the lighter sickness rates likely to be experienced in the selected societies are justified. It is, however, a known fact that these class societies have been formed in fairly large numbers, and contain many members, and the situation has, therefore, to be faced.

### An Unforeseen Difficulty.

A potent illustration is here incidentally given of the lack of wisdom in compelling all societies to use the same sickness rates for valuation purposes. A further incidental, but none the less important, result of this separation of classes will be that, as a general rule, those societies will realise surpluses whose members are in circumstances which render insurance hardly necessary, while those societies which are likely to experience large deficiencies will most probably be composed of members who are on the whole less able financially to bear the cost of sickness, and to whom, therefore, insurance is a necessity. Such a situation would seem to be converse to that which equity demands. In addition to the dangers of over-insurance, emphasised by Sir Gerald Ryan in his speech reported last week, there are many other factors operating which will materially assist in bringing about excess sickness claims unless considerable care is taken. The malingerer has already been sufficiently discussed, so we do not deal with him here.

There is another unforeseen direction, however, in which societies are experiencing difficulty in connection with the payment of sickness claims. Under the Act the approved societies are relieved of payment of sickness benefit in all cases of compensated accident, and where in the judgment of the society a member has a right to compensation and does not obtain it, the society is entitled to be represented in the Court on his behalf. The result of this is that the Workmen's Compensation Insurance Societies, which frequently hitherto refrained from contesting doubtful cases on account of the difficulty of recovering costs, are now not so diffident when the workman has his approved society behind him. As a result many cases which would before have been paid are now contested, and the effect is naturally injurious to the approved societies, whose position is extremely difficult.

